# Effect of Biological Denitrification Inhibitor on N_2_O Emissions from Paddy Soil and Microbial Mechanisms

**DOI:** 10.3390/microorganisms13061232

**Published:** 2025-05-27

**Authors:** Longfei Wang, Kaikuo Wu, Furong Xiao, Ping Gong, Yan Xue, Yuchao Song, Ruizhuo Wang, Zhijie Wu, Lili Zhang

**Affiliations:** 1 Institute of Applied Ecology, Chinese Academy of Sciences, Shenyang 110016, China; m15036937173@163.com (L.W.); xiaofurong0911@163.com (F.X.); gongping@iae.ac.cn (P.G.); xueyanchina@163.com (Y.X.); songyuchao@iae.ac.cn (Y.S.); wuzj@iae.ac.cn (Z.W.); 2 University of Chinese Academy of Sciences, Beijing 100049, China; 3 CAS Key Laboratory of Forest Ecology and Silviculture, Institute of Applied Ecology, Chinese Academy of Sciences, Shenyang 110016, China; 4 College of Horticulture, Shenyang Agricultural University, Shenyang 110016, China; wrz329@126.com

**Keywords:** biological denitrification inhibitor, proanthocyanidins, N_2_O emissions, northeast China paddy soil, nitrate reductase activity, denitrifiers

## Abstract

The denitrification process is the main process of the soil nitrogen (N) cycle in paddy fields, which leads to the production of large amounts of nitrous oxide (N_2_O) and increases N loss in paddy soil. Plant-derived bio denitrification inhibitor procyanidins are thought to inhibit soil denitrification, thereby reducing N_2_O emissions and soil N loss. However, the denitrification inhibition effect of procyanidins in paddy soils with high organic matter content remains unclear, and their high price is not conducive to practical application. Therefore, this study conducted a 21-day incubation experiment using low-cost proanthocyanidins (containing procyanidins) and paddy soil with high organic matter content in Northeast China to explore the effects of proanthocyanidins on N_2_O emissions and related microorganisms in paddy soil. The results of the incubation experiment showed that the application of proanthocyanidins in paddy soil in Northeast China could promote the production of N_2_O in the first three days but inhibited the production of N_2_O thereafter. Throughout the incubation period, proanthocyanidins inhibited the enzyme nitrate reductase (NaR) activity and the abundance of *nirS* and *nirk* denitrifying bacteria, with a significant dose-response relationship. Although the application of proanthocyanidins also reduced the soil nitrate nitrogen (NO_3_^−^-N) content, the soil NO_3_^−^-N content increased significantly with increasing incubation time. In addition, the application of proanthocyanidins increased soil microbial respiration, ammonia-oxidizing archaea (AOA) *amoA* gene abundance, and soil ammonium nitrogen (NH_4_^+^-N) content. Therefore, the application of proanthocyanidins to paddy soil in Northeast China can effectively regulate denitrification. However, in future studies, it is necessary to explore the impact of proanthocyanidins on the nitrification process and use them in combination with urease inhibitors and/or nitrification inhibitors to better regulate soil N transformation and reduce N_2_O emissions in paddy soil.

## 1. Introduction

Nitrous oxide (N_2_O) is the third largest greenhouse gas in the world after carbon dioxide (CO_2_) and methane (CH_4_) [1]; however, its warming potential is 298 times that of CO_2_, and it greatly exacerbates the depletion of stratospheric ozone [2]. N_2_O is mainly produced in agricultural soils, accounting for over 60% of total N_2_O emissions [3]. To ensure food production, the agricultural system uses a large amount of nitrogen (N) fertilizer every year, which further leads to more N_2_O emissions [4]. Agricultural soils mainly produce N_2_O through microbially mediated nitrification and denitrification processes [5], with denitrification typically occurring in anaerobic environments [6]. Due to long-term flooding in paddy soils, denitrification has become the main pathway for N_2_O production, reaching over 77% [7], and has become an important N loss pathway, which is not conducive to sustainable agricultural development. Therefore, reducing N_2_O emissions from paddy fields has become a focus of attention for researchers [8,9,10].

The use of fertilizers containing inhibitors is one of the commonly used fertilization measures in paddy fields, which is beneficial for reducing N_2_O emissions and improving the nitrogen fertilizer use efficiency of rice [11,12]. Currently, the most commonly used inhibitors are urease and nitrification inhibitors. Urease inhibitors can slow down the hydrolysis of urea into ammonium nitrogen (NH_4_^+^-N) by inhibiting urease activity, while nitrification inhibitors can inhibit the rate of NH_4_^+^-N conversion to nitrate nitrogen (NO_3_^−^-N) [8,11,13]. However, urease and nitrification inhibitors cannot directly regulate the denitrification process; therefore, they cannot effectively regulate the N_2_O produced by denitrification [7,14]. For a long time, research has focused on reducing agriculture-related pollution through the control of nitrification, while denitrification has received comparatively little attention [5,8,11]. In recent years, Bardon et al. [15,16] discovered B-type procyanidins, a biological denitrification inhibitor (BDI) found in the roots of the invasive species *Fallopia* spp., which have inhibitory effects on soil denitrification and can also limit the loss of gaseous N in soil by directly targeting N_2_O sources. This novel B-type procyanidin has the potential to regulate denitrification in paddy soils.

Soil denitrification is an important process in soil N cycling. The first step is the reduction of NO_3_^−^-N to nitrite nitrogen (NO_2_^−^-N) by the enzyme nitrate reductase (NaR) [17]. The *nirS* and *nirK* genes, which encode the nitrite reductase enzyme (NiR), are used to indicate denitrifying bacteria that convert NO_2_^−^-N to NO. Since NO_2_^−^-N reduction is the rate-limiting step of denitrification, the functional genes *nirS* and *nirK* are often used as biomarkers to explore denitrifying bacterial communities [18,19]. Procyanidins may mainly inhibit denitrification by affecting NaR activity and the abundance of denitrifying bacteria containing *nirS* and *nirK* [17,20,21]. Previous studies have shown that soils with high organic matter content may limit the denitrification inhibition effect of procyanidins, as procyanidins can be adsorbed by soil organic matter (SOM) [22]. However, soils with high moisture content and organic matter are often more conducive to denitrification [14]. Therefore, it is necessary to test the denitrification inhibition effect of procyanidins on paddy soils with high SOM content in Northeast China.

B-type procyanidins in proanthocyanidins are considered key substances for inhibiting denitrification [16]; however, their high cost limits their application [23]. The use of proanthocyanidins containing B-type procyanidins can reduce costs by more than 2000 times (Macklin, Shanghai, China). Moreover, B-type procyanidins may have synergistic effects with other proanthocyanidins, affecting denitrification inhibition [16]. In addition, the application of proanthocyanidins to soil can introduce a large amount of carbon. When a large amount of N fertilizer is applied to paddy soil, the addition of carbon sources may lead to increased microbial activity and may have an impact on the nitrification process (another important process of N_2_O production), which is mainly regulated by the ammonia-oxidizing archaea (AOA) *amoA* and ammonia-oxidizing bacteria (AOB) *amoA* genes [19]. Therefore, exploring the effects of proanthocyanidins in paddy soil is beneficial for developing N fertilizer management measures and formulating effective N_2_O emission reduction strategies.

The aim of this study is to investigate the denitrification inhibition effect of proanthocyanidins on paddy soil with high SOM content in Northeast China. The main objectives of this study are to (i) evaluate the effect of proanthocyanidins on N_2_O emissions from paddy soil, (ii) evaluate the effect of proanthocyanidins on NH_4_^+^-N and NO_3_^−^-N in paddy soil, and (iii) evaluate the inhibitory effect of proanthocyanidins on NaR, *nirS*, and *nirK* genes.

## 2. Materials and Methods

### 2.1. Soil Properties and Experimental Design

The soil used for the incubation experiment was obtained from the Shenyang Experimental Station of the Institute of Applied Ecology, Liaoning Province, China (41°32′ N, 123°23′ E). In October 2024, soil from the paddy field at a depth of 0–20 cm was collected, naturally air-dried, mixed, sieved through a 2 mm sieve, and stored in a refrigerator at 4 °C until the incubation experiment was carried out. The basic physicochemical properties of the soil are listed in Table 1.

Before starting the incubation experiment, the soil sample was adjusted to 40% water holding capacity (WHC) and then pre-incubated for 7 days at 25 °C in an incubator to activate soil microbial activity. The pre-incubation soil was filled into 120 mL glass bottles (diameter: 4 cm, height: 9.5 cm), each containing 20 g of soil (dry soil). Four treatments were set up for the incubation experiment: no urea control check (CK), addition of urea (U), addition of urea and 1 mg of proanthocyanidins g^−1^ dry soil (U+P1), and addition of urea and 2 mg of proanthocyanidins g^−1^ dry soil (U+P2). Each treatment was repeated 3 times, totaling 84 bottles (divided into 7 destructive samples). The amount of urea added was 0.084 mg N g^−1^ dry soil. Proanthocyanins are commercial proanthocyanidins extracted from grape seeds (Macklin, Shanghai, China; proanthocyanidin content > 95%, C_30_H_26_O_13_, mainly containing gallic acid, procyanidin B1, catechin, procyanidin B2, epicatechin, epicatechin 3-O-gallate, and oligomeric proanthocyanins). After adding urea and proanthocyanidins, 27 mL of deionized water was added to form a water layer of about 5 mm in the bottle to simulate a flooded paddy field environment. All glass bottles were opened and incubated at 25 °C in an incubator until sampling, during which the evaporated water was replenished daily using the weighing method.

### 2.2. Gas and Soil Sample Collection and Determination

Gas and soil samples were collected on the 1st, 3rd, 5th, 7th, 10th, 14th, and 21st days after the start of the incubation experiment. At 8:00, 35 mL of gas was collected from the bottle using a 35 mL syringe, and the glass bottle was immediately sealed with a rubber stopper. At 10:00, another 35 mL of gas was collected from the bottle and analyzed (N_2_O and CO_2_) using a gas chromatograph (Agilent 7890B, Gas Chromatography, Wilmington, DE, USA). The N_2_O and CO_2_ emission rates were calculated based on the slope of the concentration changes of the two collected gases [12]. The cumulative emissions of N_2_O and CO_2_ were calculated using the interpolation method [24]. After each gas sample collection, the NH_4_^+^-N and NO_3_^−^-N contents and NaR activity in the soil were measured. Soil NH_4_^+^-N and NO_3_^−^-N were extracted using a 2 mol L^−1^ KCl solution (soil:solution = 1:5), filtered through filter paper, and analyzed using a continuous flow analyzer (AA3, Bran+Luebbe, Norderstedt, Germany) [24]. The activity of soil NaR was determined using α-naphthylamine-sulfanilic acid, and the specific method was described by Ye et al. [17].

Soil samples were collected on the 1st, 3rd, 5th, and 7th day after the start of the incubation experiment to determine N cycling functional genes. The abundance of AOA *amoA*, AOB *amoA*, *nirS*, and *nirK* was determined using real-time quantitative PCR (qPCR) on an ABI 7300 system (Applied Biosystems, Waltham, MA, USA). The primers and qPCR thermal profiles are shown in Table S1. The reaction mixture contained 10 µL 2×Taq Plus Master Mix, 0.8 µL primers, 1 µL DNA template, and 7.4 µL deionized water. All qPCR reactions were performed using melting point curve analysis to determine the amplified products. Three parallel qPCR replicates were performed.

### 2.3. Statistical Analyses

Data analysis was conducted using one-way ANOVA in SPSS Statistics 16.0 (SPSS Inc., Chicago, IL, USA) (*p* < 0.05). Origin 8.5 software (Origin Lab Corp., Northampton, MA, USA) was used to create the figures. Data are presented as mean ± standard error (SE).

## 3. Results

### 3.1. Effects of Proanthocyanidins on N_2_O Production and Microbial Respiration

**Figure 1:**
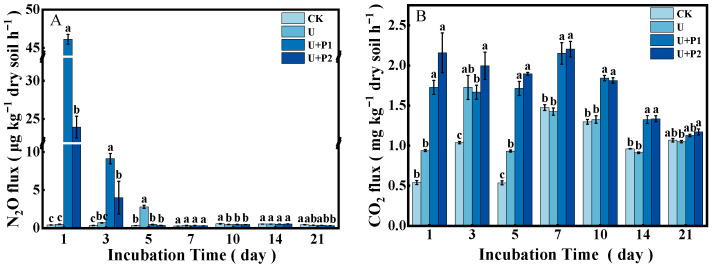
Effect of proanthocyanidin application on N_2_O (**A**) and CO_2_ (**B**) fluxes in paddy soil. No urea control check (CK), addition of urea (U), addition of urea and 1 mg of proanthocyanidins g^−1^ dry soil (U+P1), and addition of urea and 2 mg of proanthocyanidins g^−1^ dry soil (U+P2). Bars represent the mean ± standard error (*n* = 3). Different lowercase letters within the treatments indicate significant differences (*p* < 0.05).

**Figure 2:**
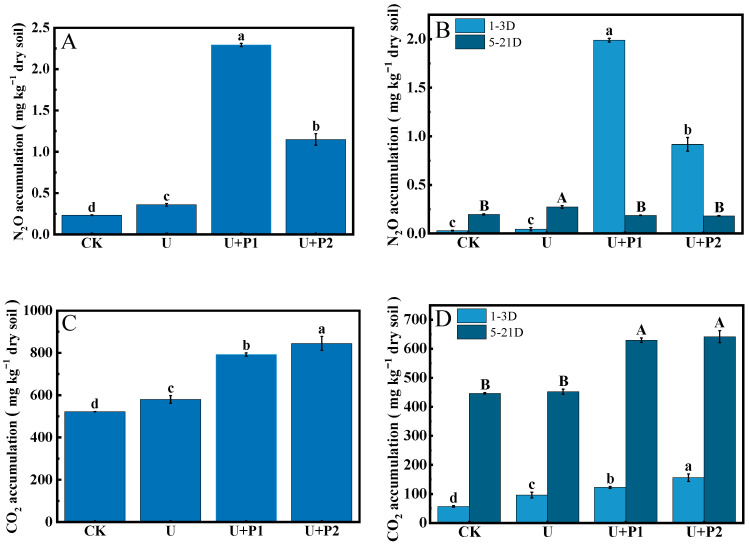
Effect of proanthocyanidin application on N_2_O ((**A**): N_2_O accumulation during the entire incubation period; (**B**): N_2_O accumulation in 1–3 days and 5–21 days) and CO_2_ ((**C**): CO_2_ accumulation during the entire incubation period; (**D**): CO_2_ accumulation in 1–3 days and 5–21 days) accumulation in different treatments. No urea control check (CK), addition of urea (U), addition of urea and 1 mg of proanthocyanidins g^−1^ dry soil (U+P1), and addition of urea and 2 mg of proanthocyanidins g^−1^ dry soil (U+P2). Bars represent the mean ± standard error (*n* = 3). Different uppercase and lowercase letters within the treatments indicate significant differences (*p* < 0.05).

During the incubation experiment, the N_2_O flux ranged from 0.28–47.25 µg kg^−1^ dry soil h^−1^ (Figure 1A). The N_2_O flux of CK was always at a low level, and the N_2_O flux of U reached a peak on the 5th day (2.80 µg kg^−1^ dry soil h^−1^) and then declined rapidly. The N_2_O fluxes of U+P1 and U+P2 peaked on the 1st day at 46.11 and 23.94 µg kg^−1^ dry soil h^−1^, respectively, and then declined rapidly thereafter. As shown in Figure 1A, N_2_O produced by U+P1 and U+P2 treatments mainly occurred in the first 3 days of the incubation experiment, while N_2_O produced by U treatment mainly occurred on the 5th day. The N_2_O accumulation was shown as U+P1 > U+P2 > U > CK, and the differences were significant (*p* < 0.05), being 2.29, 1.15, 0.36, and 0.23 mg kg^−1^ dry soil, respectively (Figure 2A). In the first 3 days of the incubation experiment, N_2_O accumulation was in the order U+P1 > U+P2 > U > CK, with values of 1.99, 0.94, 0.04, and 0.03 mg kg^−1^ dry soil, respectively (Figure 2B). Except for U and CK, there were significant differences among the other treatments (*p* < 0.05, Figure 2B). During the 5–21 days of the incubation experiment, the accumulation of N_2_O showed the following order: U > CK > U+P1 > U+P2, with concentrations of 0.27, 0.20, 0.19, and 0.18 mg kg^−1^ dry soil, respectively (Figure 2B). Among these, the U treatment was significantly higher than the other three treatments (*p* < 0.05, Figure 2B).

In this study, the CO_2_ flux ranged from 0.49 to 2.65 mg kg^−1^ dry soil h^−1^ (Figure 1B). The CO_2_ fluxes of all treatments showed a trend of first increasing and then decreasing (Figure 1B). The CO_2_ flux of U peaked on day 3 (1.73 mg kg^−1^ dry soil h^−1^), while the CO_2_ flux of CK, U+P1, and U+P2 peaked on day 7 (1.48, 2.15, and 2.20 mg kg^−1^ dry soil h^−1^, respectively) (Figure 1B). As shown in Figure 2C, CO_2_ accumulation showed the order of U+P2 > U+P1 > U > CK, with significant differences among different treatments (*p* < 0.05), which were 844.59, 791.98, 579.71, and 522.08 mg kg^−1^ dry soil, respectively. In the first 3 days of the incubation experiment, the CO_2_ accumulation was in the order U+P2 > U+P1 > U > CK, with values of 154.44, 122.18, 95.93, and 56.83 mg kg^−1^ dry soil, respectively, and the differences among all treatments were significant (*p* < 0.05, Figure 2D). During 5–21 days of incubation, the CO_2_ accumulation showed that U+P2 > U+P1 > U > CK, with concentrations of 641.79, 629.22, 451.89, and 446.35 mg kg^−1^ dry soil, respectively. Among them, the U+P1 and U+P2 treatments were significantly greater than the CK and U treatments (*p* < 0.05, Figure 2D).

### 3.2. Influence of Proanthocyanidins on NaR

**Figure 3:**
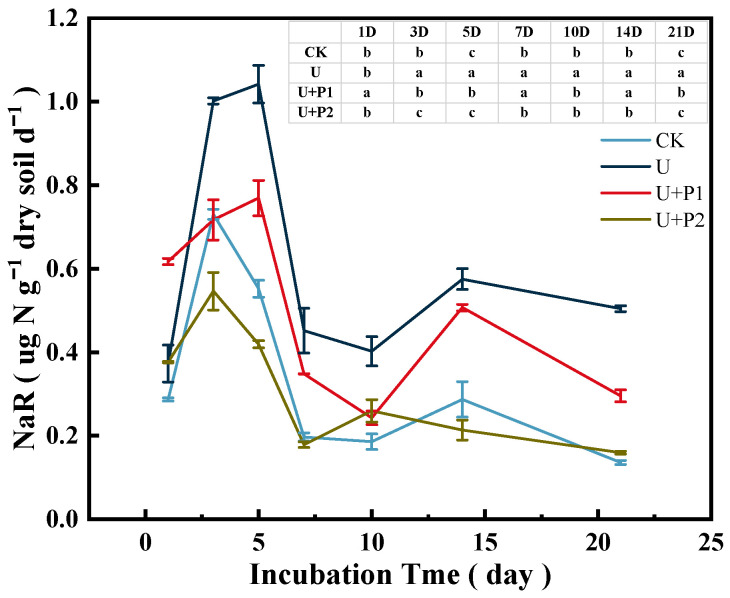
Effect of proanthocyanidin application on soil NaR activity. No urea control check (CK), addition of urea (U), addition of urea and 1 mg of proanthocyanidins g^−1^ dry soil (U+P1), and addition of urea and 2 mg of proanthocyanidins g^−1^ dry soil (U+P2). Bars represent the mean ± standard error (*n* = 3). Different lowercase letters in the table indicate significant differences (*p* < 0.05).

As shown in Figure 3, soil NaR activity during the incubation experiment ranged from 0.16–1.12 µg N g^−1^ dry soil d^−1^, showing a trend of first increasing and then decreasing. CK and U+P2 treatments peaked on day 3 at 0.73 and 0.55 µg N g^−1^ dry soil d^−1^, respectively. U and U+P1 treatments peaked on day 5 at 1.04 and 0.77 µg N g^−1^ dry soil d^−1^, respectively. Proanthocyanidins significantly inhibited soil NaR activity (*p* < 0.05), and the inhibitory effect was stronger with increasing proanthocyanidins dosage. When the application amount of proanthocyanidins was 2 mg g^−1^ dry soil, the soil NaR activity was consistent with that of the CK treatment.

### 3.3. Effects of Procyanidins on NH_4_^+^-N and NO_3_^−^-N in Paddy Soil

**Figure 4:**
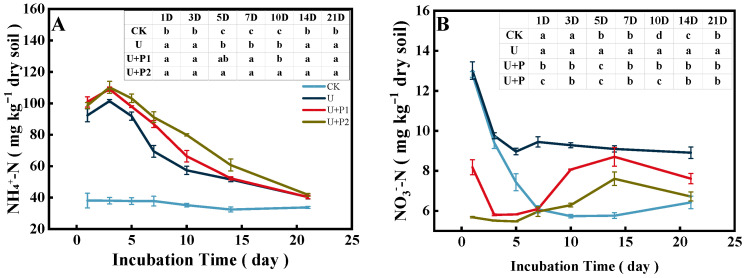
Effect of proanthocyanidin application on soil NH_4_^+^-N (**A**) and NO_3_^−^-N (**B**). No urea control check (CK), addition of urea (U), addition of urea and 1 mg of proanthocyanidins g^−1^ dry soil (U+P1), and addition of urea and 2 mg of proanthocyanidins g^−1^ dry soil (U+P2). Bars represent the mean ± standard error (*n* = 3). Different lowercase letters in the table indicate significant differences (*p* < 0.05).

The soil NH_4_^+^-N content ranged from 30.17–117.57 mg kg^−1^ (Figure 4A). Except for the CK treatment, the soil NH_4_^+^-N content in the other three treatments reached a peak on the 3rd day and then decreased rapidly, and there was no significant difference in soil NH_4_^+^-N content on the 21st day. Compared with U, the soil NH_4_^+^-N content was higher after proanthocyanidins were added, and U+P2 promoted soil NH4+-N content more than U+P1 (*p* < 0.05).

The soil NO_3_^−^-N content in the paddy soil ranged from 5.43 to 13.62 mg kg^−1^ (Figure 4B). With an increase in incubation time, soil NO_3_^−^-N content decreased rapidly in CK and U and tended to be stable, while in U+P1 and U+P2, it first decreased, then increased, and then decreased. After the application of proanthocyanidins, soil NO_3_^−^-N content increased rapidly after the 5th day and reached its peak value on the 14th day, but the soil NO_3_^−^-N content was significantly lower than that of the U treatment (*p* < 0.05).

### 3.4. Abundance of N-Transforming Functional Genes

**Figure 5:**
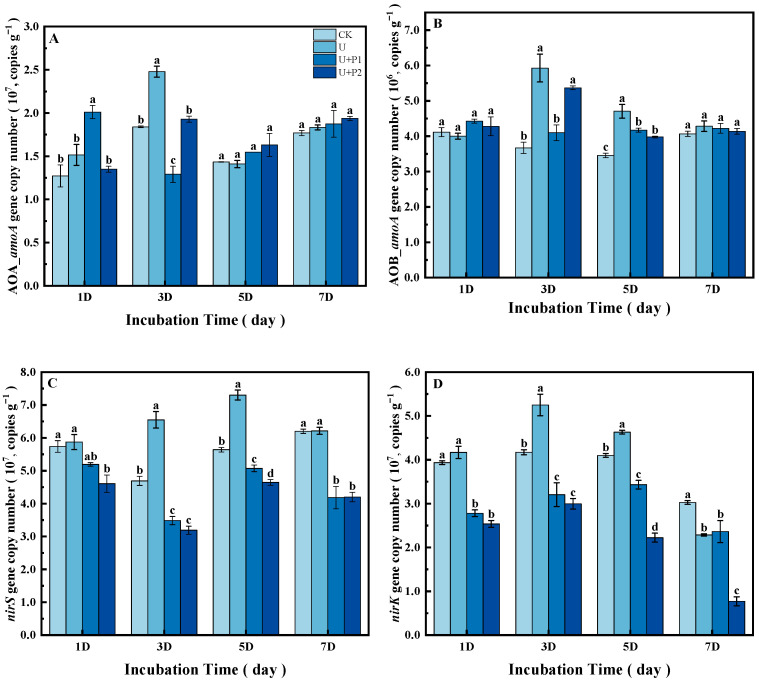
Effect of proanthocyanidin application on the abundance of AOA *amoA* (**A**), AOB *amoA* (**B**), *nirS* (**C**), and *nirK* (**D**) genes in soil. No urea control check (CK), addition of urea (U), addition of urea and 1 mg of proanthocyanidins g^−1^ dry soil (U+P1), and addition of urea and 2 mg of proanthocyanidins g^−1^ dry soil (U+P2). Bars represent the mean ± standard error (*n* = 3). Different lowercase letters within the treatments indicate significant differences (*p* < 0.05).

On the first day of the incubation experiment, the abundance of the AOA *amoA* gene in the U+P1 treatment was significantly higher than that in the other three treatments (*p* < 0.05, Figure 5A). On day 3, the abundance of the AOA *amoA* gene in the U treatment was the highest and significantly higher than that in the other three treatments (*p* < 0.05), while the abundance of the AOA *amoA* gene in the U+P1 treatment was the lowest and significantly lower than that in the other three treatments (*p* < 0.05). There was no significant difference in AOA *amoA* gene abundance between the treatments on days 5 and 7.

On days 1 and 7 of the incubation experiment, there was no significant difference in AOB *amoA* gene abundance among the treatments (Figure 5B). On day 3, the abundance of the AOB *amoA* gene in the U and U+P2 treatments was significantly higher than that in the CK and U+P1 treatments (*p* < 0.05). On day 5, the abundance of the AOB *amoA* gene was the highest in the U treatment, followed by U+P1 and U+P2 treatments, and the lowest in the CK treatment (*p* < 0.05).

Urea application (U) significantly increased the abundance of *nirS* genes on days 3 and 5 of the incubation experiment (*p* < 0.05, Figure 5C). The addition of proanthocyanidins significantly inhibited the abundance of *nirS* genes, and there was a significant dose-response relationship; that is, the inhibitory effect was enhanced with an increase in dosage (*p* < 0.05, Figure 5C).

Similarly, urea application (U) significantly increased *nirK* gene abundance on days 3 and 5 of the incubation experiment but decreased *nirK* gene abundance on day 7 (*p* < 0.05, Figure 5D). The addition of proanthocyanidins significantly inhibited the abundance of the *nirK* gene and also had a significant dose-response relationship, especially on days 5 and 7 of the incubation experiment (Figure 5D).

### 3.5. Relationship of N_2_O Flux to Soil Properties and Microorganisms

**Figure 6:**
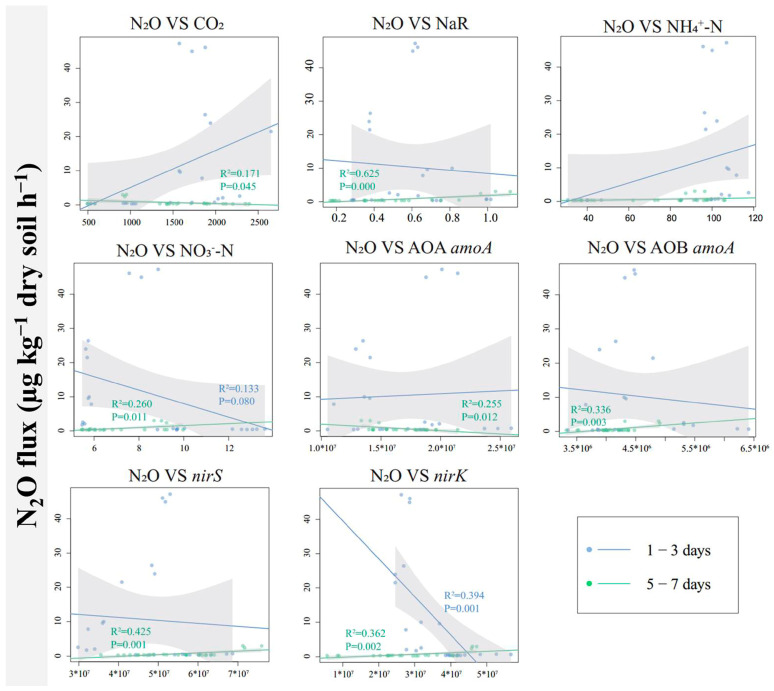
The linear regression relationship between N_2_O flux and microbial respiration, NaR activity, soil NH_4_^+^-N, soil NO_3_^−^-N, or between N_2_O flux and the abundance of functional genes related to N_2_O emissions. Only those with a correlation of *p* < 0.05 are listed. The shaded area represents a 95% confidence interval.

Linear regression was used to explore the potential relationships between N_2_O flux and soil properties, NaR activity, and the abundance of associated functional genes (Figure 6). There was a significant negative correlation between N_2_O flux and *nirK* gene abundance on days 1–3 of the incubation experiment. On days 5–7, N_2_O flux was negatively correlated with microbial respiration (CO_2_ flux) and AOA *amoA* gene abundance but positively correlated with soil NaR activity, soil NO_3_^−^-N content, AOB *amoA* gene abundance, *nirS* gene abundance, and *nirK* gene abundance.

## 4. Discussion

In this study, the addition of urea significantly promoted N_2_O emissions, which is consistent with previous studies [3,6,10]. The addition of urea promoted soil microbial activity (Figure 1B, with a significant increase in CO_2_ emissions), NaR activity (Figure 3) and increased the abundance of ammonia-oxidizing and denitrifying bacterial communities, including ammonia-oxidizing archaea and ammonia-oxidizing bacteria containing *amoA*, as well as denitrifying bacteria containing *nirS*- and *nirK*- (Figure 5). AOA *amoA*, AOB *amoA*, and denitrifying bacteria containing *nirS*- and *nirK*- are closely related to soil N_2_O production [25,26].

Compared with urea application alone, the addition of proanthocyanidins in the first three days of the incubation experiment promoted the production of N_2_O in paddy soil, which was inconsistent with a previous study in which the addition of procyanidins inhibited the production of N_2_O in soil [14,27]. This may be due to several reasons.

(1) The paddy soil used in this study was in a flooded state, and proanthocyanidins have extremely strong water solubility [15], which may have weakened their denitrification inhibition ability. In our study, we also found that on the first day of the incubation experiment, the NaR activity of the U+P1 treatment was higher than that of the U treatment (Figure 3), and the soil NO_3_^−^-N content of U+P1 decreased rapidly from 1 to 3 days of the incubation experiment (Figure 4B), while the inhibitory effect of U+P2 on NaR activity was better than that of U+P1 (Figure 3). Therefore, the inhibitory effect of proanthocyanidins on soil denitrification shows a dose-response relationship; that is, the application of proanthocyanidins in paddy soil may require higher doses.

(2) Oxygen was still present in the soil pores during the early stage of the incubation experiment. Soil heterogeneity allows for the coexistence of aerobic and anaerobic regions [28], resulting in nitrification being an important process for the generation of N_2_O in addition to denitrification [7,29]. The addition of proanthocyanidins, due to the input of a large amount of activated carbon, stimulated the nitrification process to produce more N_2_O. In this study, the addition of proanthocyanidins promoted the abundance of AOA *amoA*, which supported this point. Starting from the 7th day of the incubation experiment, all treatments had extremely low and almost no difference in N_2_O emissions, which may be due to the decrease in NO_3_^−^-N content and O_2_ depletion, where N_2_O acts as an electron acceptor and is reduced to N_2_ [23,29]. Therefore, the application of proanthocyanidins in paddy fields may need to be performed at an appropriate time (for example, three days after flooding).

(3) The proanthocyanidins used in this study not only contain procyanidins but also easily decomposable carbon sources, such as gallic acid, catechins, and epicatechins. At low concentrations, these substances have almost no antibacterial activity [16] and may promote microbial growth (Figure 1B, with a significant increase in CO_2_ emissions). This intensified urea decomposition, providing more substrates for the nitrification process (Figure 4A, with a significant increase in soil NH_4_^+^-N content), thereby promoting the generation of N_2_O [30]. Therefore, considering the economic cost, more effective proanthocyanidins should be screened, and it is necessary to further explore the contributions of different pathways (four pathways: nitrifier nitrification (NN), nitrifier denitrification (ND), nitrification-coupled denitrification (NCD), and heterotrophic denitrification (HD)) to N_2_O production under the application of proanthocyanidins [29], which is conducive to formulating more reasonable application strategies for proanthocyanidins, such as combined application with urease inhibitors and/or nitration inhibitors.

Starting from the fifth day of the incubation experiment, the addition of proanthocyanidins significantly inhibited the N_2_O emissions. This might be due to oxygen depletion and denitrification in the paddy soil [29]. The addition of proanthocyanidins inhibited NaR activity (Figure 3) and the abundance of *nirS* and *nirK* genes (Figure 5). While NaR activity, the abundances of *nirS* and *nirK* genes were highly positively correlated with N_2_O production (Figure 6, [17]), thus reducing N_2_O production.

Throughout the entire incubation period, the addition of proanthocyanidins resulted in higher soil NH_4_^+^-N content and lower soil NO_3_^−^-N content than the application of urea alone. This might be because the application of proanthocyanidins inhibited denitrification, enhanced the immobilization of soil NO_3_^−^-N by microorganisms [31], and the process of dissimilatory NO_3_^−^-N reduction to NH_4_^+^-N (Figure 4A, [32]), and ultimately increased soil N [17]. After the 5th day of the incubation experiment, the soil NO_3_^−^-N content in the paddy soil with the addition of proanthocyanidins increased significantly. This might be due to the fact that the soil denitrification process dominated during this period [29], while proanthocyanidins inhibited soil denitrification, resulting in an increase in soil NO_3_^−^-N concentration [23,33].

## 5. Conclusions

Our study shows that the application of proanthocyanidins to paddy soils in Northeast China can inhibit soil denitrification, primarily by inhibiting NaR activity and the growth of *nirS*- and *nirK-type* denitrifying bacteria. The inhibitory effect of proanthocyanidins on soil denitrification was dose-dependent; that is, the inhibitory effect of soil denitrification was enhanced by an increase in the amount of proanthocyanidins. In addition, proanthocyanidins can increase the soil NH_4_^+^-N content and N_2_O production in the first three days of the culture test and promote the abundance of the AOA *amoA* gene, which may be related to the nitrification process of paddy soil. Therefore, in future field practices, proanthocyanidins should be applied in combination with urease and nitration inhibitors, and the application time of proanthocyanidins should be determined to achieve more effective field N management. This study is helpful for the application of proanthocyanidins in paddy fields.

## Figures and Tables

**Table 1:** Soil physicochemical properties (0–20 cm soil layer).

Soil Type	Organic C (g kg^−1^)	Total N (g kg^−1^)	NH_4_^+^-N (mg kg^−1^)	NO_3_^−^-N (mg kg^−1^)	Available P (mg kg^−1^)	Rapidly Available K (mg kg^−1^)	pH
Alfisol	13.18	1.24	6.83	10.86	9.24	82.66	6.84

## Data Availability

All relevant data are included in this article.

## Supplementary Materials

The following supporting information can be downloaded at: https://www.mdpi.com/article/10.3390/microorganisms13061232/s1, Table S1: Primer sequences of some key N cycling genes used for real time PCR [5,34,35].
