# Supplementary material for: Effect of Biological Denitrification Inhibitor on N2O Emissions from Paddy Soil and Microbial Mechanisms
Source: Microorganisms. 2025 May 27;13(6):1232. doi: 10.3390/microorganisms13061232 (PMC12195389; doi:10.3390/microorganisms13061232)
Supplement: Supplementary file 1 [file microorganisms-13-01232-s001.zip › microorganisms-3622372-supplementary.pdf]

# Supplementary Information (SI)

**Table S1** Primer sequences of some key N cycling genes used for real time PCR

| Target Gene | Primer | Primer Sequence 5'–3' | Thermal Profile | Reference |
|---|---|---|---|---|
| AOA *amoA* | Arch-amoAF | STAATGGTCTGGCTTAGACG | 94°C for 2 min followed by 40 cycles of 45 s at 94°C, 1 min at 53°C, 45 s at 68°C, plate read at 83°C | [1] |
| | Arch-amoAR | GCGGCCATCCATCTGTATGT | | |
| AOB *amoA* | amoA–1F | GGGGTTTCTACTGGTGGT | 95°C for 5 min followed by 35 cycles of 45 s at 95°C, 45 s at 55°C and 1 min at 72°C | [1] |
| | amoA–2R | CCCCTCKGSAAAGCCTTCTTC | | |
| *nirS* | nirS cd3a F | GTSAACGTSAAGGARACSGG | 95°C for 2 min × 1 cycle; 95°C for 45 s, 55°C for 45 s, 72°C for 45 s, 85°C for 20 s × 40 cycles | [2] |
| | nirS R3cd R | GASTTCGGRTGSGTCTTGA | | |
| *nirK* | F1aCu | ATCATGGTSCTGCCGCG | 95°C for 3 min × 1 cycle; 95 °C for 30 s, 58 °C for 40 s, 72 °C for 40 s, 83 °C for 15 s × 40 cycles | [3] |
| | R3Cu | GCCTCGATCAGRTTGTGGTT | | |

**References**


1. Shen, J.P.; Zhang, L.M.; Zhu, Y.G.; Zhang, J.B.; He, J.Z. Abundance and composition of ammonia-oxidizing bacteria and ammonia-oxidizing archaea communities of an alkaline sandy loam. *Environ. Microbiol.* **2008**, 10, 1601-1611. https://doi.org/10.1111/j.1462-2920.2008.01578.x
2. Di, H.J.; Cameron, K.C.; Podolyan, A.; Robinson, A. Effect of soil moisture status and a nitrification inhibitor, dicyandiamide, on ammonia oxidizer and denitrifier growth and nitrous oxide emissions in a grassland soil. *Soil Biol. Biochem.* **2014**, 73, 59-68. https://doi.org/10.1016/j.soilbio.2014.02.011
3. Wang, Q.; Hu, H.W.; Shen, J.P.; Du, S.; Zhang, L.M.; He, J.Z.; Han, L.L. Effects of the nitrification inhibitor dicyandiamide (DCD) on $N_2O$ emissions and the abundance of nitrifiers and denitrifiers in two contrasting agricultural soils. *J. Soil. Sediment.* **2016**, 17, 1635-1643. https://doi.org/10.1007/s11368-016-1633-9
